# Innate lymphocyte cells in asthma phenotypes

**DOI:** 10.1186/s13601-015-0068-5

**Published:** 2015-07-06

**Authors:** Leyla Pur Ozyigit, Hideaki Morita, Mubeccel Akdis

**Affiliations:** Department of Allergy and Immunology, Koç University, School of Medicine, Istanbul, Turkey; Swiss Institute of Allergy and Asthma Research, University of Zurich, Zurich, Switzerland; Christine Kühne-Center for Allergy Research and Education, Davos, Switzerland

**Keywords:** Asthma, Innate immunity, Airways, Phenotype, Cytokines

## Abstract

T helper type 2 (T_H_2) cells were previously thought to be the main initiating effector cell type in asthma; however, exaggerated T_H_2 cell activities alone were insufficient to explain all aspects of asthma. Asthma is a heterogeneous syndrome comprising different phenotypes that are characterized by their different clinical features, treatment responses, and inflammation patterns. The most-studied subgroups of asthma include T_H_2-associated early-onset allergic asthma, late-onset persistent eosinophilic asthma, virus-induced asthma, obesity-related asthma, and neutrophilic asthma. The recent discovery of human innate lymphoid cells capable of rapidly producing large amounts of cytokines upon activation and the mouse data pointing to an essential role for these cells in asthma models have emphasized the important role of the innate immune system in asthma and have provided a new means of better understanding asthma mechanisms and differentiating its phenotypes.

## Introduction

The immune system is classically divided into two categories, innate and adaptive immunity, according to the speed and the duration of the response, and they collaborate with each other to target different agents and perform effector functions. Through recent advances in understanding the different subsets of immune system effector cells, Annunziato et al. have recently suggested a new classification [1]. They proposed that the innate and adaptive immune systems could also be generally classified into three major kinds of cell-mediated effector immunity: categorized as type 1, comprising T-bet^+^ IFN-γ–producing helper cells, type 2, composed of GATA-3^+^ lymphocytes producing interleukin-4 (IL-4), IL-5, and IL-13, and type 3, characterized by RORγt^+^ lymphocytes that produce IL-17 alone or in combination with IL-22 as signature cytokines [1].

Innate immunity is known to respond quickly and without antigen specificity to signals derived from the environment or from other immune cells. Innate lymphoid cells (ILCs) are the newest described elements of the innate immune system and have received much attention over the last few years [2]. Early in the immune response, ILCs possess a lymphoid morphology, similar to adaptive B and T cells, and produce many different T helper (T_H_) cell cytokines but lack the recombination-activating gene (RAG)-mediated antigen specific receptors; therefore, these cells are not antigen-specific. Because ILCs are very similar to the other effector cell phenotypes, it was proposed that ILCs could be classified in a similar manner to that of T_H_ cells. Type 1 immunity includes the IFN-γ-producing group 1 ILCs (ILC1s) that cope with intracellular pathogens through activation of mononuclear phagocytes. Group 2 ILCs (ILC2s), which secrete IL-4, IL-5, IL-9, and IL-13, are an example of Type 2 immunity. This type of immunity induces mast cell, basophil, and eosinophil activation leading to an increase in serum IgE levels and, therefore, fosters the eradication of helminthes and venoms. Group 3 ILCs (ILC3s), which are an example of type 3 immunity, produce IL-17 and/or IL-22, activate mononuclear phagocytes, recruit neutrophils, and induce epithelial antimicrobial responses, all of which help protect against extracellular fungal and bacterial infections [1]. This group includes lymphoid tissue inducer (LTi) cells that promote the formation of lymph nodes [3].

In general, ILCs constitute a distinct element of the innate immune system, providing an initial host response via specific cytokines after sensing external stimuli on the frontline. The initial priming of immune responses to pathogenic challenges is executed by ILCs with the capacity to rapidly secrete effector cytokines. All ILCs are developmentally related, and they all require the expression of the transcriptional repressor inhibitor of DNA binding 2 (Id2) and the common IL-2 cytokine receptor (γ_c_) chain. Moreover, they all possess the IL-7 receptor α-chain (CD-127) [4].

The ILC lineage incorporates the classic cytotoxic natural killer (NK) cells and the non-cytotoxic ILC family [5]. Natural killer cells are also capable of responding to invading pathogens and exterior threats without the need for prior sensitization, and they function in the absence of RAG-recombined antigen receptor recognition. Beside their ability to release a variety of cytokines, they also have the capacity to kill other cells. NK cells were initially categorized into ILC1s, but recently it has been shown that these cells are different from non-cytotoxic ILCs because they undergo different developmental pathways [6, 7].

Non-cytotoxic ILCs have the capacity to rapidly respond to the environment by producing various cytokines, and their goal is to maintain homeostasis with tissue repair and remodeling. They are involved in lymphoid organ development and in resistance to pathogenic and nonpathogenic microorganisms. Non-cytotoxic ILCs also interact with mast cells, natural killer T (NKT) cells, eosinophils, epithelial cells, and macrophages, and they may configure the optimal milieu for setting up an adaptive response [8, 5].

Asthma includes complex innate and adaptive immune responses to environmental factors. For decades, researchers investigating the immune responses in asthma have focused on adaptive immunity, mostly on memory responses to antigens. Therefore, asthma was previously considered to be the airway manifestation of a T_H_2-driven response from adaptive immunity toward some specific triggers [9]. Today, advances in molecular technology and recent immunology studies have allowed us to understand much more about the impact of the innate immune system on the development of asthma and on its evolution. Negative results from the initial monoclonal treatment drug studies and cluster analysis have demonstrated that “asthma syndrome” covers distinct subgroups of a reversible obstructive lung disease with different clinical properties termed different “phenotypes” [10–12]. Although there is no consensus on a single phenotype classification for asthma, the most-studied subgroups include: T_H_2-associated with early-onset allergic asthma, late-onset persistent eosinophilic asthma, virus-induced asthma, obesity-related asthma, and neutrophilic asthma. All of these subgroups can be distinguished from each other by clinical factors, such as the patient age at disease onset and the involvement of particular biological pathways.

Understanding new innate pathways will allow for more accurate asthma phenotyping and, subsequently, will help direct us to personalized care for our asthmatic patients. In this review, we provide an updated view on the emerging roles of non-cytotoxic ILCs in different asthma phenotypes.

## Review

### ILC1s and its possible role in asthma phenotypes

ILC1s, formerly known as conventional NK cells, are present in mucosal tissues, express the IL-7 receptor, and rapidly secrete IFN-γ upon stimulation with IL-12 and IL-18, which are produced by macrophages and other cells. ILC1s are involved in the antiviral response and have been shown to expand in the intestines of patients with Crohn’s disease [13]. Although we now know that NK cells are developmentally different from ILC1s and that ILC1s lack cytotoxicity, these two cell types share some common properties [14]. Therefore, it is postulated that, like NK cells in a mouse model [15] and in human asthmatics [16], ILC1s might also have a role in the development of eosinophilic airway inflammation, which can be seen in most asthma phenotypes and even in the microbiota–immune interactions of asthma [17]. Intraepithelial ILC1s, another subset of ILC1s, have been found in human tonsillar tissue [18]. Unlike typical ILC1s, these cells are not stimulated with IL-12 and IL-18, but rather with IL-15.

### ILC2s and early onset allergic asthma

For many years, early onset allergic asthma has been considered to be an adaptive immune response that develops after the prior sensitization phase to allergens. Airway epithelial cells are the frontline cells initially exposed to inhaled substances, and they actively collaborate with other immune cells, specifically pulmonary dendritic cells (DC) followed by M2 macrophages, to mount a T_H_2 response through the production of epithelial cell-derived cytokines, such as IL-33, IL-25 and thymic stromal lymphopoietin (TSLP) [8].

After recent studies questioning the requirement for antigen-specific adaptive T_H_ cells in allergic asthma, the existence of a new class of the innate type-2 lymphocyte group, the ILC2s, has been described. ILC2s were first observed in the gut, emphasizing their physiological role against helminth infection [19–21]. Later, their presence was confirmed in various other tissues, including in the human lung [22]. ILC2s are also present in human peripheral blood, and their percentage is greater in asthma patients than it is in allergic rhinitis patients or in healthy controls [23, 24].

**Fig. 1:**
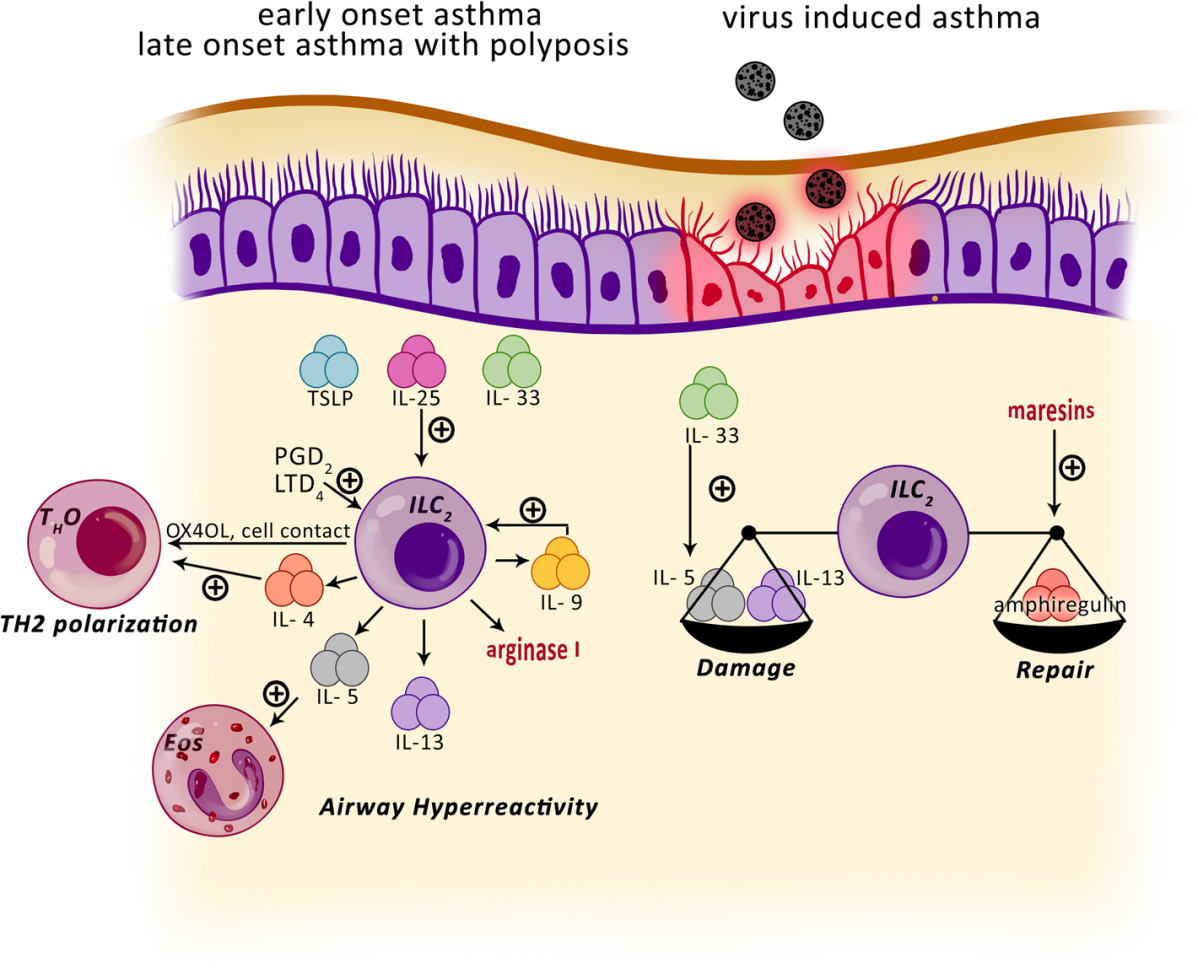
Function and regulation of group 2 lymphoid cells in different asthma phenotypes. Innate lymphoid cells group 2 (ILC2s) of early onset asthma and late onset asthma with polyposis are regulated by several elements such as the epithelial cell derived thymic stromal lymphopoietin (TSLP), interleukin 25 (IL-25) and IL-33; arachidonic acid metabolites, like prostaglandin D_2_ (PGD2) and leukotriene D_4_ (LTD_4_). Lung ILC2s produces IL-9 that also regulates their activation. ILC2s release IL-4, IL-5 and IL-13; then increase the airway hyperreactivity and eosinophilia. Lung ILC2s also secrete arginase 1. ILC2s can stimulate naive T cells (TH0) by IL-4, costimulatory molecules OX40L and a contact-dependent mechanism favoring T_H_2 polarization. In the virus induced asthma phenotype, lungs ILC2s constitute a balance between tissue repair and tissue damage via amphiregulin and type 2-cytokine secretion. The damage is potentialized by IL-33 and the repairing capacity is enhanced by maresins. Eos, eosinophil

Following contact with certain microbial products, helminth infection, physical injury, or allergens in the airway, epithelial cells secrete TSLP, IL-25, and IL-33 [25, 26, 23, 19]. Afterwards the recruitment and activation of innate type 2 cells can initiate the immune response independently of adaptive immunity [27–29]. Lung ILC2s are an important source of IL-5, a growth and differentiation factor for eosinophils, and of IL-13, which can directly cause airway hyperreactivity (AHR). Cytokine production is followed by a progressive accumulation of eosinophils and mucus secretion. IL-13 is also crucial for the differentiation of T_H_2 cells from naive CD4+ T cells (Fig. 1) [22, 21, 20, 30]. Mouse studies have demonstrated a role for ILC2s in OVA-, HDM-, papain protease-, and *Alternaria alternata*-induced airway inflammation [31, 29, 32, 22, 33, 34]. Some of these observations are from RAG-deficient animals, which are adaptive immunity-deficient mice. Although evidence supporting this in human asthma has not been found yet, we speculate that the activation of ILC2s in the absence of T cells and B cells is enough to induce asthma-like symptoms, and that ILC2s may play a role in early onset allergic asthma.

A papain-induced asthma model showed that even in the presence of T cells, ILC2s were the major source of type 2 cytokines [22]. Another mouse model with papain-induced airway inflammation revealed that lung ILCs also produce IL-9, depending on the amount of IL-2 from the adaptive immune system, and IL-33 [35]. Moreover, a recent study showed that ILC2s in the lungs secrete arginase-1, a key enzyme in the pathophysiology of acute and chronic allergic asthma (Fig. 1) [36–38].

Being at the side that first contacts the environment, as well as the first source of type 2 cytokines, it is likely that ILC2s have a role in preparing a type 2 milieu for setting up the adaptive immune response [8]. Furthermore, major histocompatibility complex II (MHCII) is expressed on ILC2s, which provides them with the capacity for antigen presentation [39, 20]. ILC2s can promote the effector functions of CD4+ T cells via costimulatory molecules OX40L and IL-4 and by a contact-dependent mechanism favoring T_H_2 polarization [40, 41]. Mutually-activated ILC2s also need IL-2, possibly derived from T cells, for activation and survival [21, 20].

### ILC2s and late onset asthma with nasal polyposis

Asthma onset after 12 years of age and the presence of blood eosinophilia are two important parameters for differentiating the immunologically and pathologically distinct asthma phenotype known as late onset asthma with nasal polyposis [42]. This phenotype is frequently associated with nasal polyposis and sometimes with aspirin-sensitivity [11]. Nevertheless, allergy skin test results are often positive in asthma patients with this phenotype, and even though these patients may rarely feel that their allergy symptoms were triggered by the allergens for which they tested positive [42].

Mjösberg et al. first identified ILC2s in nasal polyps of patients with rhinosinusitis (CRSwNP) [23]. Several studies have reported an increased percentage of ILC2s in the sinus mucosa of these patients compared with that in chronic rhinosinusitis patients without nasal polyps [43–45]. IL-25, IL-33, and eotaxin-3 levels, released from the sinus mucosa epithelium were also increased in CRSwNP [46]. Additionally, these patients had upregulated IL-5 and IL-13 mRNA levels [43]. The stimulation of ILC2s from human nasal polyps with TSLP has been shown to result in IL-4 release (Fig. 1) [47]. Another report found that ILC2s frequencies were associated with tissue and blood eosinophilia [45]. Additional studies focusing on the effects of ILC2s frequency on asthma control, the severity of this phenotype, and the association with the presence of aspirin sensitivity are needed.

### ILC2s in virus-associated asthma and AHR

Viruses can pave the way for the development of asthma in susceptible individuals. After 2 years of age, viruses can be the trigger for a distinct phenotype of asthma known as “virus-induced asthma”. Moreover, viruses frequently provoke asthma exacerbations [48–51].

In an experimental mouse model, researchers have shown that influenza A virus can rapidly induce AHR by inducing the activation of ILCs independently of the adaptive immune system [52]. During influenza virus infection, IL-33 is released from alveolar macrophages and NKT cells, which induces ILC2 activation and the subsequent production of type 2 cytokines, IL-13 and IL-5 [52, 53]. The presence of IL-5 enables the growth and the later persistence of eosinophils, even after viral clearance. IL-5 and IL-13 are mainly responsible for the clinical symptoms of AHR. Consequently, ILC2s can promote inflammation, but they also have an opposing role during virus-induced AHR- specifically the repair of wounded lung tissue after virus infection. This effect is attained through amphiregulin, an epidermal growth factor-like growth factor (Fig. 1) [4]. The balance between the damage and repair of airways constitutes the homeostatic function of ILC2s.

### Regulation of ILC2s function during asthma

Recent work on ILC2s has provided new insights into T_H_2-mediated asthma phenotypes, but additional questions remain. Future studies are needed to determine how this newly found source of type 2 cytokines could be regulated and how this knowledge will ameliorate our treatment options.

#### Role of TSLP, IL-25, and IL-33 in regulating ILC2s

Human ILC2s can be stimulated by TSLP, IL-25, and IL-33 [23, 44, 22]. Intranasal administration of IL-25 or IL-33 induces an increase in cytokine-releasing ILC2s in the lungs, bronchoalveolar lavage fluid, and mediastinal lymph nodes [31, 29, 54, 55].

- IL-25 has an essential role in allergic airway inflammation and also in remodeling [56]. Neutralizing antibodies against IL-25 may prevent airway hyperresponsiveness in allergic asthma [57].
- IL-33 can also activate mast cells and basophils through IgE receptors, and is a survival factor for eosinophils [58, 59]. Its effect on ILC2s is even faster and stronger than that of IL-25 [60]. These properties make IL-33 a possible target for future therapies. Like neutralizing antibodies to IL-25, neutralizing antibodies to IL-33 or to IL-33 receptor (ST2) has been shown to reduce AHR and to lessen the eosinophilic response [61].

#### Role of specialized pro-resolving mediators (SPM)

Asthma is an inflammatory lung disease with impaired resolution mechanisms, and understanding more about immune resolution could provide new treatments for this disease. SPM, which are essential fatty acids derived from regulating molecules, possess potent anti-inflammatory and pro-resolving capacities [62, 63]. They include lipoxins, resolvins, protectins, and maresins [64]. Investigating how ILC2s can be regulated through SPM will provide new insights into asthma pathobiology and could result in new therapeutic approaches [62].

- Lipoxins are the leading family of SPM [63]. Lipoxin A_4_ might inhibit the stimulatory effects of PGD_2_, IL-25, and IL-33 [16].
- Maresins are the most recently described SPM family. In a recent study, researchers demonstrated that maresins reduce lung inflammation and ILC2s expression of cytokines and increase the repairing capacity of ILC2s through amphiregulin (Fig. 1) [65]. Furthermore, regulatory T cells (Tregs) play a mandatory role in this interaction. Therefore, as potent regulators of Tregs and ILC2s, maresins may be promising therapeutic targets for asthma.

#### Role of leukotrienes and prostaglandins

Human ILC2s are stimulated by arachidonic acid metabolites, such as leukotrienes [32] and prostaglandins [16].

- Lung ILC2s express receptors for cysteinyl leukotrienes, including cysteinyl leukotriene receptor 1 (CysLT1R), the high-affinity receptor for leukotriene D_4_ (LTD_4_). Following stimulation by LTD_4,_ ILC2s produce IL-4, IL-5, and IL-13. Montelukast, a CysLT1R antagonist, can prevent the IL-5 production stimulated by leukotriene C_4_ and LTD_4_ [32].
- Prostaglandin D_2_ (PGD2) is a positive regulator of ILC2s, inducing ILC2s migration and production of type 2 cytokines [16, 66]. PGD2 binds to its recently characterized receptor, Chemokine receptor, a homologous molecule expressed on T helper type 2 cells (CRTH2), which is a receptor expressed on ILC2s that is similar to a T_H_2 receptor [67].

Recently, a study evaluating the effect of subcutaneous grass pollen immunotherapy (SCIT) on peripheral ILC2s demonstrated that the percentage of ILC2s in untreated allergic rhinitis patients increased during pollen season, and that this percentage is correlated with the patient’s symptom scores. In contrast, the percentage of peripheral ILC2s in allergic rhinitis patients who were treated with SCIT and in control patients did not increase during pollen season [68]. An evaluation of whether this same effect occurs in allergic asthma patients remains to be conducted.

### ILC3s in non-allergic asthma

Non-T_H_2 asthma is poorly defined and is less well understood than allergic asthma phenotypes, even though it represents a large proportion of total asthma cases [11]. This group of asthma phenotypes includes obesity-associated asthma and neutrophilic asthma.

Although the role in non-allergic asthma of type 3 immunity and IL-17, which is believed to be a T_H_2-released cytokine, have only recently become an area of interest, a combination of bench and bedside approaches should improve our understanding of these phenotypes [11]. Recent studies have emphasized the role of IL-17 on steroid-resistant AHR [69, 70].

**Fig. 2:**
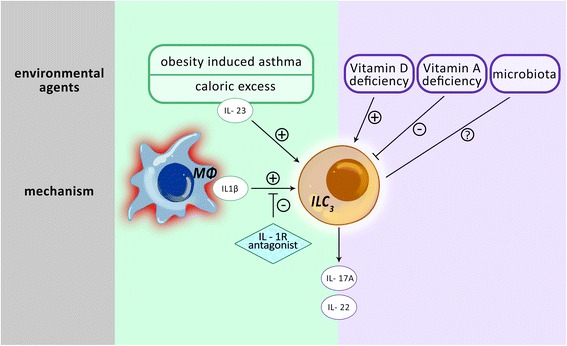
Mechanism of innate lymphoid cells group 3 in obesity induced asthma and their regulation. Innate lymphoid cells group 3 (ILC3s) produce interleukin 17A (IL-17A) and IL-22. Macrophages (Mϕ) produce IL-1β that engages IL-1 receptor on innate lymphoid cells group 3 (ILC3s) resulting in airway hyperreactivity. This effect can be inhibited by an IL-1 receptor (IL-1R) antagonist. ILC3s are sensitive to environmental factors, micronutrients and microbiota. Vitamin D deficiency increases ILC3s’ functions whereas Vitamin A deficiency leads to a reduction; the influence of airway microbiota on ILC3s is still unknown

ILC3s are mainly found in gut-associated lymphoid tissue (GALT) [71], but their presence in the lung has also been demonstrated [72]. They express MHC class II and are able to regulate the adaptive immune system by presenting antigens [73]. IL-23 and IL-1β rapidly stimulate ILC3s to produce IL-22, which plays a protective role through lung epithelial cells during T_H_2 asthma (Fig. 2) [74]. ILC3s may also produce IL-17A, which is a potent neutrophil chemotactic agent. The presence of IL-22 and IL-17A in the sputum or peripheral blood is positively correlated with the severity of asthma [75–79]. However, further studies are needed to show the role of these cytokines in non-T_H_2 asthma.

### Obesity-associated asthma

This asthma phenotype is difficult to control because of comorbidities and a lack of responsiveness to classic asthma treatments [11, 80]. In a mouse model of obesity-induced AHR, researchers showed a crucial role in AHR for IL-17A, which is secreted mainly from ILC3s in the absence of adaptive immunity. The same study was the first to report the presence of ILC3s in the bronchoalveolar lavage fluid of patients with lung diseases. The researchers also reported that patients with severe asthma had a higher percentage of lung IL-17-producing ILC3s, than patients with mild or no asthma. Surprisingly, a protective role for ILC2s, in which they maintain the metabolic homeostasis in obesity, has been recently demonstrated [81, 82]. This unexpected finding suggests that the role of ILC2s in obesity-associated asthma should be studied further.

### Regulation of ILC3s function and asthma

Although ILC3s are typically stimulated by IL-23 and IL-1β, they are also sensitive to environmental signals, such as caloric excess, micronutrients, and microbiota. A vitamin A deficit in mice resulted in greatly decreased numbers of ILC3s in the intestine, which increased the susceptibility of these mice to bacterial infections. Subsequently, treatment with vitamin A restored the number of ILC3s to normal levels; however, this treatment reduced the percentage of ILC2s [83]. In another study, vitamin D deficiency improved ILC3s responses (Fig. 2) [84]. ILCs are influenced by the ability of macrophages to sense microbial signals and produce IL-1β [85]. Interestingly, a study demonstrated that the AHR in obese mice was completely resolved with an IL-1 receptor antagonist, anakinra. The researchers also reported a decrease in the number of IL-17-producing lung ILC3s [72]. The microbiota possessed by asthmatic individuals in their airways is believed to have a higher potential to be pathogenic than that of non-asthmatic individuals [86]. How ILC3s contribute to and/or are impacted by the roles of these vitamins and the influence of this crosstalk with microbiota has not yet been evaluated.

## Conclusion

Knowledge gained from recently recognized ILCs will help us to fill in the missing gaps of innate molecular pathways regarding asthma immunopathology. The lung ILCs on the frontier, sensitive to environmental factors including toxic and non-toxic substances, pathogenic and nonpathogenic microorganisms, and allergens, maintain homeostasis with tissue repair and remodeling. They can initiate AHR and appropriately set up the milieu for adaptive immunity by producing various cytokines, generally previously described in other contexts, and by interacting with different immune cells. ILCs represent one of the very first mediators for the different phenotypes of asthma ‘syndrome*’* [10]. However, it is still unclear whether additional subsets of ILCs exist, and their role in innate immune memory has yet to be determined. We need further studies investigating their interaction with other immune cells, exogenous factors, and other micronutrients. A better understanding of their pathogenesis in asthma will be important for a better understanding of asthma phenotypes and for developing better strategies for preventive and therapeutic interventions.
