# Erratum to: Innate lymphoid cells in asthma phenotypes

**DOI:** 10.1186/s13601-015-0075-6

**Published:** 2015-09-15

**Authors:** Leyla Pur Ozyigit, Hideaki Morita, Mubeccel Akdis

**Affiliations:** Department of Allergy and Immunology, Koç University, School of Medicine, Istanbul, Turkey; Swiss Institute of Allergy and Asthma Research, University of Zurich, Zurich, Switzerland; Christine Kühne-Center for Allergy Research and Education, Davos, Switzerland

## Erratum to: Clinical and Translational Allergy (2015) 5:23 DOI 10.1186/s13601-015-0068-5

After publication of the original article, the authors noticed the title was incorrect. The title was published as: “Innate lymphocyte cells in asthma phenotypes”; however, it should be “Innate lymphoid cells in asthma phenotypes”, as seen above.

